# Foregut caustic injuries: results of the world society of emergency surgery consensus conference

**DOI:** 10.1186/s13017-015-0039-0

**Published:** 2015-09-26

**Authors:** Luigi Bonavina, Mircea Chirica, Ognjan Skrobic, Yoram Kluger, Nelson A. Andreollo, Sandro Contini, Aleksander Simic, Luca Ansaloni, Fausto Catena, Gustavo P. Fraga, Carlo Locatelli, Osvaldo Chiara, Jeffry Kashuk, Federico Coccolini, Yuri Macchitella, Massimiliano Mutignani, Cesare Cutrone, Marco Dei Poli, Tino Valetti, Emanuele Asti, Michael Kelly, Predrag Pesko

**Affiliations:** Department of Surgery, IRCCS Policlinico San Donato, University of Milan Medical School, Piazza Malan 1, 20097 San Donato Milanese (Milano), Italy; Department of Digestive Surgery, Saint-Louis Hospital, Paris, France; Department of Surgery, University of Belgrade, Belgrade, Serbia; Department of General Surgery, Rambam Health Care Campus, Haifa, Israel; Department of Surgery, University of Campinas, Campinas, Brasil; University of Parma, Parma, Italy; General Surgery I, Papa Giovanni XXIII Hospital, Bergamo, Italy; Emergency Surgery Department, Maggiore Parma Hospital, Parma, Italy; Institute of Toxicology, University of Pavia, Pavia, Italy; Emergency Department, Niguarda Hospital, Milan, Italy; Department of Surgery, University of Jerusalem, Jerusalem Rehovot, Israel; Department of Endoscopy, Niguarda Hospital, Milan, Italy; Department of Otolaryngology, Azienda Ospedaliera, Padova, Italy; Intensive Care Unit, IRCCS Policlinico San Donato, San Donato Milanese, Italy; Department of Anesthesiology, Papa Giovanni XXIII Hospital, Bergamo, Italy; Department of Surgery, Wagga Wagga Hospital, Wagga Wagga, Australia

## Abstract

**Introduction:**

Lesions of the upper digestive tract due to ingestion of caustic agents still represent a major medical and surgical emergency worldwide. The work-up of these patients is poorly defined and no clear therapeutic guidelines are available.

**Purpose of the study:**

The aim of this study was to provide an evidence-based international consensus on primary and secondary prevention, diagnosis, staging, and treatment of this life-threatening and potentially disabling condition.

**Methods:**

An extensive literature search was performed by an international panel of experts under the auspices of the World Society of Emergency Surgery (WSES). The level of evidence of the screened publications was graded using the Oxford 2011 criteria. The level of evidence of the literature and the main topics regarding foregut caustic injuries were discussed during a dedicated meeting in Milan, Italy (April 2015), and during the 3rd Annual Congress of the World Society of Emergency Surgery in Jerusalem, Israel (July 2015).

**Results:**

One-hundred-forty-seven full papers which addressed the relevant clinical questions of the research were admitted to the consensus conference. There was an unanimous consensus on the fact that the current literature on foregut caustic injuries lacks homogeneous classification systems and prospective methodology. Moreover, the non-standardized definition of technical and clinical success precludes any accurate comparison of therapeutic modalities. Key recommendations and algorithms based on expert opinions, retrospective studies and literature reviews were proposed and approved during the final consensus conference. The clinical practice guidelines resulting from the consensus conference were approved by the WSES council.

**Conclusions:**

The recommendations emerging from this consensus conference, although based on a low level of evidence, have important clinical implications. A world registry of foregut caustic injuries could be useful to collect a homogeneous data-base for prospective clinical studies that may help improving the current clinical practice guidelines.

## Introduction

A wide variety of chemical agents including mineral and organic acids and alkalis, oxidizing agents, denaturants, hydrocarbons and other chemicals may cause corrosive injuries. Although the mechanism, the severity, and the timing of the injury may vary, all these substances cause damage to living tissue on contact. Accidental or intentional ingestion of corrosive substances cause life-threatening injuries of the upper digestive tract. The degree of injury is related to the concentration, amount, viscosity, and duration of exposure to the caustic agent. The large majority of caustic agents are liquids. Strong acids and alkali are readily available as household cleaners. Lye is a generic term for the alkali used to make soap, either potassium hydroxide or sodium hydroxide. Acids cause coagulation necrosis, whereas alkali combine with tissue proteins and cause liquefaction necrosis which penetrates deep into tissues. Concentrated alkali ingestion may lead to more serious injury and complications by penetrating tissues and leading to full-thickness damage of the esophageal/gastric wall. Liquid household bleach, although often reported, does rarely cause severe injuries. Children under the age of 5 years account for more than 80 % of accidental caustic ingestion, whereas adult injuries are more often intentional and suicidal [1–3].

Foregut caustic ingestion is certainly an under-reported public health issue. Primary prevention of these dramatic injuries was initiated in the United States by Chevalier Jackson who began a campaign that led to the Federal Caustic Act (1927) which mandated proper labeling of these harmful compounds. Subsequent acts have enforced proper labeling, antidote instructions, concentration restrictions, and child-resistant packaging. The effects of these changes have decreased but not eliminated the incidence and severity of caustic ingestions in the United States [4]. Nowadays, most information on foregut caustic injuries comes from countries where legislation is less restrictive or even absent, such as Africa, Turkey, India, Eastern Europe, Southeast Asia, and France [5].

Caustic ingestion can result in a number of injuries ranging in severity from mild oral burns to minimal mucosal erythema or transmural necrosis of the esophagus and stomach with visceral perforation. Emergency surgery is indicated for hemorrhage, free perforation, mediastinitis or peritonitis. Full thickness esophagogastric necrosis is a severe form of injury associated with considerable morbidity and mortality. It may occur due to ingestion of a large amount or highly concentrated corrosive substance. The injury may extend and involve adjacent viscera such as the duodenum, small intestine, colon, pancreas, and gallbladder. Complications such as hemorrhage, perforation, aorto-enteric fistula, or gastro-colic fistula may occur in patients surviving the initial event during the first 2–3 weeks after ingestion. Patients who have survived severe caustic injury of the foregut are at high risk of luminal strictures. After recovery from the initial injury, collagen deposition and fibrosis continue for months and scar retraction results in esophageal shortening and stricture. The incidence of esophageal stricture following grade IIB and grade III esophageal burns is in the range of 50–80 % [6, 7]. Concomitant gastric outlet obstruction occurs in up to 30 % of patients with esophageal stricture [8, 9]. In the long term, development of pharyngeal, esophageal, or gastric strictures may compromise nutritional outcome. Interestingly, the risk of squamous-cell cancer of the damaged esophagus is estimated to be 1000 times higher than that of the general population, and the latent period for the malignant change is 15–40 years [10, 11].

Currently, in most referral centers therapeutic algorithms for the management of patients with caustic injuries rely on the findings of upper digestive endoscopy. Despite the use of different endoscopic classification systems, therapeutic approaches are similar and include conservative management of patients with mild injuries, while patients with severe injuries undergo emergency surgical exploration. Although there are studies describing the short and long-term outcomes of reconstruction for established caustic strictures of the esophagus, there is limited literature on the early and long term outcomes of patients managed in an emergency setting for corrosive induced acute esophagogastric and/or adjacent organ necrosis.

## Methods

Two independent MEDLINE and EMBASE searches were performed to identify relevant papers published between 1990 and 2015. The following medical subject headings terms were used in the searches: caustic ingestion, caustic lesions, corrosive injuries, esophagus, stomach, esophageal dilatation, gastric outlet obstruction. The search terms were identified in the title, abstract, or medical subject heading. Initially, 2143 abstracts of the retrieved studies were reviewed and screened for exclusion criteria. At the end of the search, 1113 abstracts that fulfilled the inclusion criteria were selected. Finally, 147 full papers which addressed the relevant clinical questions of the research were admitted to the consensus conference. The level of evidence for each recommendation statement was assigned by using the grading system proposed by the Oxford Centre for Evidence-Based Medicine [12]. A preliminary manuscript was prepared by an international panel of 12 experts including anesthesiologists, endoscopists, surgeons, and toxicologists. The key recommendations and algorithms were discussed at a dedicated meeting held in Milan in April 2015, and at the 3rd Congress of the World Society of Emergency Surgery held in Jerusalem in July 2015. Finally, evidence based guidelines for the management of foregut caustic injuries were developed to outline clinical recommendations.

### Recommendations

#### Initial therapeutic approach [13–57]

##### Pre-hospital management

- Establish the diagnosis of caustic agent ingestion. Identify the involved agent. Collect the product on the scene and bring it to the emergency department. If difficult product identification try to evaluate pH (<2; > 10), but be aware that some agents cause a pH-independent corrosive injury. (Level 4–5)
- Evaluate the ingestion scenario by: a) ascertain ingestion; b) determine the accidental or voluntary character; c) detect co-ingestion of alcohol and/or drugs; d) try to evaluate ingested quantity (in adults assess normal sip (30–50 ml) vs. large gulp (60–90 ml); e) assess delay from ingestion. (Level 4–5)
- Identification of additional risk factors such as extreme ages (young children, elderly), pregnancy, underlying disease, and the form of the ingested agent (solid, liquid, gel, vapors-concomitant aspiration). (Level 5)
- Supportive care rather than specific antidotes is the mainstay of treatment. Secure airway patency and hemodynamic stabilization. Prevent vomiting, repeat esophageal passage, and aspiration by: a) use of antiemetics (metoclopramide); b) seated 45° position during transport; c) avoid gastric lavage and induced emesis; d) avoid diluents (milk, water). (Level 5)
- Avoid increasing damage by exothermic reaction: attempts at pH neutralization with either a weak alkali or acid are prohibited. (Level 5)

##### Emergency department management

- Continue symptomatic treatment including adequate pain relief while waiting to evaluate the severity of caustic injuries. If airway support is required favor fiberoptic laryngoscopy over blind intubation; perform tracheotomy if necessary. (Level 5)
- Laboratory tests should include WBC, hemoglobin, platelet count, CRP, pH, and serum levels of Na, K, Cl, Ca, Mg, urea, creatinin, LDH, CPK, AST, ALT, lactates, alcohol. β-HCG levels should be measured in young women. (Level 5)
- Contact Poison Control Centers to evaluate systemic toxicity of the ingested agents. (Level 4–5)
- Avoid nasogastric tube positioning as their validity to prevent vomiting and stricture formation has never been proven; nasogastric tubes have been reported to increase risks of gastric perforation, gastroesophageal reflux, and pneumonia. (Level 5)
- The efficacy of proton-pump inhibitors and H2 blockers in minimizing esophageal injury by suppressing acid reflux has not been proven. (Level 5)
- The utility of corticosteroid in terms of stricture prevention is controversial and systematic administration is not recommended. Steroids should be reserved for patients with symptoms involving the airway. (Level 3)
- Administration of broad-spectrum antibiotics should not be done on a systematic basis. Antibiotics are advised in grade 3 injuries if corticosteroids are initiated or if lung involvement is identified. (Level 5)

##### Management algorithm

**Fig. 1:**
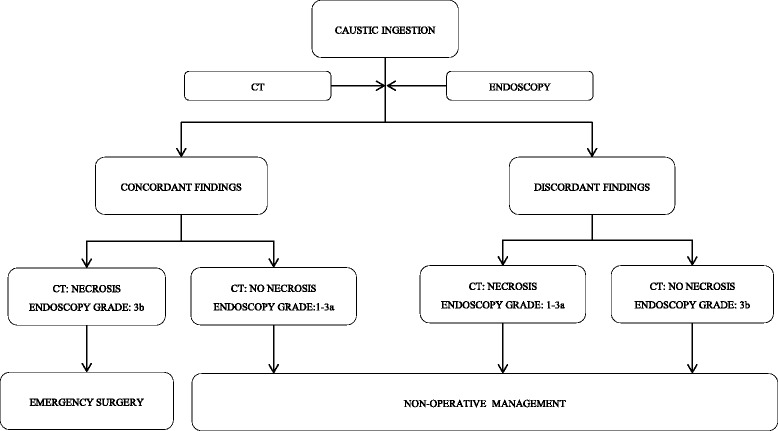
Management algorithm for caustic ingestion based on computed tomography and endoscopic findings

- Patients with clinical signs of peritonitis and hemodynamic instability require immediate surgical exploration. Although symptoms such as chest pain, dysphagia, odynophagia, drooling, hemorrhage are usually associated with severe injuries after voluntary ingestion, the absence of oropharyngeal damage does not exclude the possibility of severe esophagogastric injuries. (Level 4)
- Results of laboratory tests such as WBC, platelet count, CRP, pH, AST, ALT, creatinin, and lactate can help decision making in difficult situations. (Level 5)
- Endoscopy is the cornerstone of management of caustic injuries. Endoscopy is usually performed 3 to 6 h after ingestion, and injuries are graded according to the Zargar classification. Patients with severe (grade 3b) esophagogastric injuries are considered for surgery while patients with low grade injuries (≤ grade 3a) are offered non-operative treatment. Endoscopy grading can also predict the risk to develop an esophageal stricture during follow-up. Inability of endoscopy to predict accurately the depth of intramural necrosis may result in either futile surgery, with negative effects on survival, digestive function and management costs, or in patient death due to inappropriate non operative treatment. Moreover, emergency endoscopy is futile in up to 30 % of patients who do not have injuries of the upper digestive tract following ingestion of bleach or corrosive agents other than strong acids or alkali. (Level 4)
- Computed tomography helps palliate shortcomings of endoscopy based algorithms. The use of CT is helpful in guiding indications for esophagectomy in patients with grade 3b caustic injury (Fig. 1). In a recent study CT did better than endoscopy in selecting patients for surgery or non-operative treatment, suggesting that CT can replace endoscopy in the management of caustic injuries. CT criteria of transmural esophageal necrosis include esophageal-wall blurring and periesophageal-fat blurring on unenhanced images, and absence of post-contrast esophageal-wall enhancement; transmural necrosis of the stomach was defined as the absence of post-contrast gastric-wall enhancement. (Level 3)

##### Emergency surgery for caustic injuries

- Emergency surgery is eventually required in a small number of patients with transmural necrosis to avoid involvement of adjacent organs and death. Laparotomy is usually performed but laparoscopic exploration has been reported as feasible and safe. (Level 4)
- Transhiatal esophagectomy and total gastrectomy are the most frequently employed surgical procedures in the acute setting. Meanwhile, esophagectomy with gastric preservation and total gastrectomy with esophagojejunostomy can be performed if transmural necrosis is limited to the esophagus or the stomach, respectively. (Level 4)
- Feeding jejunostomy should be systematically constructed at the end of the operation, regardless of the type of surgical procedure performed. (Level 4)
- Extended surgery (beyond esophagogastrectomy) should be attempted in case of existing injuries on other abdominal organs. All injured organs should be resected during the first operation as caustic lesions invariably progress. Mortality rates are high, but surgery may be only choice for these patients. (Level 4)
- If the patient’s conditions allow, immediate biliary and pancreatic reconstruction should be attempted after pancreatoduodenectomy for caustic necrosis. (Level 5)
- Transmural esophageal necrosis may lead to subsequent tracheobronchial extension in a small number (<10 %) of patients. Preoperative bronchoscopy should be performed in all patients considered for surgery. In the presence of tracheobronchial necrosis, esophagectomy should be performed by a right thoracic approach. Tracheobronchial necrosis can be successfully treated with pulmonary patch technique. (Level 4)
- Massive intestinal necrosis should be a reason for the surgeon to stop due to inability of later reconstruction and nutrition. (Level 5)
- Mortality rates are high, but surgery may be only choice for these patients. Factors which have a negative impact on outcome include advanced age, tracheobronchial injuries, emergency esophagectomy, need for extended resections and severe modifications of laboratory tests (pH < 7.2, AST > 2 N, renal failure, etc.). (Level 4)
- The need to perform emergency surgery for caustic injuries has a persistent long-term negative impact both on survival and functional outcome. Moreover, esophageal resection is an independent negative predictor of survival after emergency surgery. (Level 4)

##### Nutritional approach

- Caustic ingestion can induce SIRS or sepsis with a severe hypermetabolic and catabolic response. Negative nitrogen balance and weight loss are related to injury severity. (Level 3–4)
- Use as soon as possible the gastrointestinal tract for nutrition. Patients with low grade injuries should resume oral alimentation as soon as they are able to swallow. In patients with severe burns, enteral feeding through jejunostomy or nasojejunal tube is recommended rather than a gastrostomy due to the possibility of a hidden gastric outlet obstruction (Level 5)

#### Endoscopic treatment of esophageal stricture [58–98]

- Esophageal caustic strictures are frequently complex, i.e. long (>2 cm), angulated, irregular, and multiple. In addition, the “remodeling time”, i.e. the time to stricture stabilization ranges between 6 months to 3 years. As a consequence, the reported success rate of dilatation is lower than for other benign esophageal strictures. The optimum time for dilatation is after healing of the acute injury, usually in the 3rd week. Late management is usually associated with marked esophageal wall fibrosis and collagen deposition, which requires more endoscopic sessions for adequate dilatation. This is a crucial issue in developing countries, where late presentations are more than 50 %. (Level 3–4)
- Dilatation can be carried out with balloon dilators or Savary bougies. A prospective randomized study has shown no clear advantage of each method in peptic esophageal strictures. Savary dilators are considered more reliable and effective than balloon dilators in consolidated strictures. Moreover, Savary bougies offer the advantage of “feeling” the resistance to dilatation under the operator’s hands. (Level 4)
- “Rules of the thumb”: 1)To begin with dilators that are one or two French sizes smaller than the estimated diameter of the stricture, 2)Not to dilate more than two to three sizes larger than the size of the first dilator meeting resistance. (Level 4)
- The perforation rate after dilatation for caustic strictures ranges from 0.4 to 32 %, higher than for other benign strictures. Fluoroscopy during dilatation may help in difficult cases. Although comparative trials are not available, selective use of fluoroscopy is supported by extensive clinical experience. (Level 4)
- The interval between dilatations varies from less than 1 week to 2–3 weeks. Although three or four sessions may provide durable results, the number of dilatation required is unpredictable and the endoscopic treatment may continue for years. A cut-off value for stopping dilatations is not clear and is influenced by patient, physician and geographic factors. In adult studies, the maximal esophageal wall thickness at CT scan and the involvement of muscolaris propria at endoscopic ultrasound were found to be significant predictors of stricture development, more difficult dilatations, and recurrent stricture. Gastroesophageal reflux and alterations in esophageal motility due to esophageal wall fibrosis can contribute to persistent dysphagia, in spite of apparently successful dilatations. (Level 4)
- A sustained esophageal lumen patency is not the only therapeutic goal: especially in children, an associated improvement in nutritional status should be considered an important end-point. (Level 3–4)
- Gastrostomy feeding may be life-saving, especially in developing countries. Moreover, gastrostomy allows a retrograde approach for dilatation. In challenging strictures, a nylon thread left between the nose and the gastrostomy maintains luminal access and facilitates further dilatations. (Level 3–4)
- Use of intralesional steroids and Mitomycin C applied endoscopically have been evaluated in several studies. Apparently, intralesional steroids favour a longer symptom-free time interval between dilatations but seem less effective in caustic strictures. Overall, there is no convincing evidence of the efficacy of these procedures. (Level 5)
- There is no convincing evidence that intraluminal stenting as an alternative to repeat dilatations is beneficial. Early stenting has been proposed to prevent stricture in uncontrolled studies. The number of dilatations and the duration of treatment were reduced. Notably, 50 % of children in whom a home-made stent was placed after the first dilatation, did not require further treatment. (Level 5)
- Long-term outcomes of stent placement for refractory benign esophageal strictures are poor. Partially covered SEMS are almost abandoned, in spite of their superior anchoring capacity, because of the associated hyperplastic ingrowth or overgrowth, with consequent difficult removal and recurrent dysphagia. Fully covered Self Expanding Polyflex Stents (SEPS) and Self Expanding Metallic Stents (SEMS) show a reduced reactive hyperplasia at a price of a higher migration rate. Biodegradable (BD) stents begin to degrade after 4–5 weeks and to dissolve within 2–3 months. Although the migration rate is reduced owing to the uncovered design, BD stents are only temporarily effective and sequential stenting has been suggested to avoid serial dilations. Moreover, hyperplastic tissue reactions have emerged as a significant problem. A recent systematic review of patients with a benign esophageal stricture (25 % caustic) treated by SEPS showed that only 52 % of the patients were dysphagia-free after a median follow-up of 13 months. A recent publication evaluated the results of stenting in benign strictures from twelve prospective studies. The stent was successfully placed in 98.7 %, but the overall clinical success rate was 24.2 %. (Level 4)

#### Management of gastric outlet obstruction [99–118]

- Grade three injury to the stomach is an independent risk factor for gastric outlet obstruction that can occur from a few days up to 6 years after caustic ingestion. Distal obstructions account for 60–100 % of the lesions and are located in the prepyloric area. Endoscopic balloon dilatation is safe and successful in the management of a subgroup of patients with gastric outlet obstruction. Progressive endoscopic dilatation can safely be initiated even at 2 weeks from ingestion, especially in short strictures (<25 mm). (Level 3–4)
- There is no clear evidence supporting the use of stents in the management of gastric outlet obstruction. (Level 5)
- Early surgery seems to decrease morbidity and mortality, but elective surgery earlier than 3 months is considered risky because of the poor nutritional status, adhesions, edematous gastric wall, difficult assessment of the extent of gastric resection due to ongoing fibrosis. (Level 5)
- Pyloroplasty may be performed in patients with moderate/localized strictures, but the risk of recurrent stricture is high. (Level 5)
- Gastrojejunostomy is indicated in the presence of extensive perigastric adhesions, unhealthy duodenum, and poor general condition; marginal ulcerations are reported. In selected patients the operation can safely be performed through a laparoscopic approach. (Level 5)
- The indication for gastric resection as prophylaxis against malignancy has been probably overstated in the literature. Partial gastric resection seems preferred by most surgeons. The type of surgery should be chosen according to local and general conditions. (Level 5)

#### Esophageal reconstruction [119-156]

- When esophageal dilatation is not possible or fails to provide an adequate esophageal caliber in the long-term, esophageal replacement by retrosternal stomach or, preferably, colonic interposition should be considered. (Level 3–4)
- A laryngoscopic examination is mandatory prior to all esophageal reconstructions for caustic injuries. (Level 4–5)
- The surgical bypass should be performed at least 6 months after caustic ingestion or emergency surgery since the “remodeling time”, i.e. time to stricture stabilization, is rather long. (Level 3–4)
- Removal of the native esophagus in adult patients is largely debated. It seems advisable in children because of the higher risk of cancer in the long-term. (Level 5)
- No randomized studies address the issue of which type of esophagoplasty is preferable. There are pros and cons for either right or left colon. An expert surgeon should do what he/she is used to do. (Level 5)
- One-stage esophageal resection and replacement with a gastric conduit, instead of a bypass, is feasible and safe in patients with isolated distal esophageal strictures. (Level 5)
- Minimally invasive/hybrid surgical techniques have been used with favourable results in selected patients. (Level 5)
- Angiographic study of the vascular pedicle is not routinely recommended before colon interposition or bypass, with the exception of patients with previously failed surgical attempts. (Level 5)
- Surgical revision is effective in patients who present with redundancy of the interposed colon years after retrosternal or mediastinal reconstruction. (Level 4)
- Pharyngeal strictures are difficult to manage and require special expertise. Endoscopic laser therapy of pharyngo-laryngeal adhesions may prove useful in selected patients before definitive surgical treatment. Colopharyngoplasty for strictures involving the pharynx is a safe and effective procedure. In such circumstances, the restoration of upper digestive tract continuity requires concomitant esophageal and pharyngeal reconstruction with resection of all scar tissue. Treatment of pharyngeal and laryngeal injuries should be done at the same surgical session. Supraglottic laryngectomy and suprahyoid pharyngectomy are required if the epiglottis and/or the base of the tongue are involved (Level 4–5)
- Temporary tracheostomy is mandatory during the rehabilitation training period after colopharyngoplasty. The postoperative re-education process is long and difficult and requires full cooperation from a psychiatric stable patient. (Level 5)
- Advanced age has a negative impact on esophageal reconstruction. Patients older than 55 years are likely to experience severe complications, worse functional outcomes, and decreased long-term survival. For these reasons colopharyngoplasty should not be offered after this age limit. (Level 4)
- Use of myocutaneous flaps and free jejunal grafts should be considered for salvage cervical esophageal reconstruction and restoration of alimentary transit after previously failed surgical attempts. (Level 4)

## Conclusions

The recommendations of these clinical practice guidelines are based on an extensive review of the literature and expert advice. Published data lack homogeneous classification systems and prospective methodology, the majority of papers being retrospective case reports, case series, or literature reviews. Moreover, the non-standardized definition of technical and clinical success precludes any accurate comparison of therapeutic modalities. There has been only one negative controlled trial assessing the effect of steroids to prevent esophageal stricture after caustic ingestion in children [157]. For all the above reasons, the extrapolated recommendations are mainly based on expert opinions, retrospective studies, and literature reviews with a low level of evidence.

Despite all these limitations, the value of this consensus conferences was to gather a panel of recognized experts and over 200 physicians attending the two meetings. Among the debated issues, an unanimous consensus has emerged on the fact that use of CT scan in the initial patient staging may indeed represent a true change of paradigm. The new diagnostic algorithm may allow to avoid endoscopy in selected patients, to increase the rate of esophageal preservation, and it could translate into a better long-term patients outcome and quality of life. Interestingly, a previous WSES survey has found that 80 % of the responders to a questionnaire treat fewer than ten cases of caustic ingestion per year [158], indicating the utility to apply and share clinical practice guidelines to improve patients’ care.

Caustic ingestion continues to be a complex clinical problem and a burden for the healthcare systems worldwide. Emergency surgery and subsequent alimentary tract reconstruction are a formidable challenge in these patients. On the light of the consensus that has emerged among experts in Milan and Jerusalem, the World Society of Emergency Surgery will be running a World Registry of foregut caustic injuries (www.clinicalregisters.org). This could be the first step toward a more standardized data collection and the implementation of prospective clinical studies.

## References

[1] Sarfati E, Gossot D, Assens P, Celerier M (1987). Management of caustic ingestion in adults. Br J Surg.

[2] Kikendall JW (1991). Caustic ingestion injuries. Gastroenterol. Clin. N. Am.

[3] Hugh TB, Kelly MD (1999). Corrosive ingestion and the surgeon. J Am Coll Surg.

[4] Leape LL, Ashcraft KW, Scarpelli DG, Holder TM (1971). Hazard to health-liquid lye. N Engl J Med.

[5] Contini S, Scarpignato C (2013). Caustic injury of the upper gastrointestinal tract: a comprehensive review. World J Gastroenterol.

[6] Mehta SK, Mehta S, Nagi B, Kochlar R, Zargar SA (1992). Ingestion of strong corrosive alkalis: spectrum of injury to upper gastrointestinal tract and natural history. Am J Gastroenterol.

[7] Cheng HT, Cheng CL, Lin CH, Tang JH, Chu YY, Liu N (2008). Caustic ingestion in adults: the role of endoscopic classification in predicting outcome. BMC Gastroenterol.

[8] Peracchia A, Bardini R, Bonavina L, Pavanello M, Asolati M, Buin F (1989). Lesioni da caustici dell’esofago. Esperienza su 143 casi osservati. Chirurgia.

[9] Knezevic JD, Radovanovic NS, Simic AP, Kotarac MM, Skrobic OM, Konstantinovic VD (2007). Colon interposition in the treatment of esophageal caustic strictures: 40 years of experience. Dis Esophagus.

[10] Imre J, Kopp M (1972). Arguments against long-term conservative treatment of oesophageal strictures due to corrosive burns. Thorax.

[11] Appelqvist P, Salmo M (1980). Lye corrosion carcinoma of the esophagus: a review of 63 cases. Cancer.

[12] OCEBM Levels of Evidence Working Group. The Oxford 2011 levels of evidence. htpp://www.cebm.net/index.aspx?o=5653.

[13] Andreoni B, Farina ML, Biffi R, Crosta C (1997). Esophageal perforation and caustic injury: emergency management of caustic ingestion. Dis Esophagus.

[14] Benjamin B, Agueb R, Vuarnesson H, Trachart H, Munoz Bongard N, Sarfati E (2015). Chirica M.

[15] Berthet B, Castellani P, Brioche MI, Assadourian R, Gauthier A (1996). Early operation for severe corrosive injury of the upper gastrointestinal tract. Eur J Surg.

[16] Boybeyi O, Karnak I, Tanyel FC, Senocak ME (2009). Management of unusually extensive esophagogastric corrosive injuries: emergency measures and gastric reconstruction. J Pediatr Surg.

[17] Braga M, Ljungqvist O, Soeters P, Fearon K, Weinmann A, Bozzetti F (2009). ESPEN guidelines on parenteral nutrition: Surgery. Clin. Nutr.

[18] Bonnici KS, Wood DM, Dargan PI (2014). Should computerised tomography replace endoscopy in the evaluation of symptomatic ingestion of corrosive substances?. Clin. Toxicol. (Phila.).

[19] Cabral C, Chirica M, de Chaisemartin C, Gornet JM, Munoz-Bongrand N, Halimi B (2012). Caustic injuries of the upper digestive tract: a population observational study. Surg Endosc.

[20] Cattan P, Munoz Bongrand N, Berney T, Halimi B, Sarfati E, Celerier M (2000). Extensive abdominal surgery after caustic ingestion. Ann Surg.

[21] Celik B, Nadir A, Sahin E, Kaptanoglu M (2009). Is esophagoscopy necessary for corrosive ingestion in adults?. Dis Esophagus.

[22] Ozokutan BH, Gündüz F, Gözen A, Ceylan H (2011). Gastric perforation after corrosive ingestion. Pediatr Surg Int.

[23] Chiba S, Brichkov I (2014). Pulmonary Patch Repair of Tracheobronchial Necrosis With Perforation Secondary to caustic ingestion. Ann Thorac Surg.

[24] Chirica M, Kraemer A, Petrascu E, Vuarnesson H, Pariente B, Halimi B (2014). Esophagojejunostomy after total gastrectomy for caustic injuries. Dis Esophagus.

[25] Chirica M, Resche-Rigon M, Munoz Bongrand N, Zohar S, Halimi B, Gornet JM (2012). Surgery for caustic injuries of the upper gastrointestinal tract. Ann Surg.

[26] Chirica M, Resche-Rigon M, Pariente B, Fieux F, Sabatier F, Loiseaux F (2015). Computed tomography evaluation of high-grade esophageal necrosis after corrosive ingestion to avoid unnecessary esophagectomy. Surg Endosc.

[27] Chirica M, Vuarnesson H, Zohar S, Faron M, Halimi B, Munoz Bongrand N (2012). Similar outcomes after primary and secondary esophagocoloplasty for caustic injuries. Ann Thorac Surg.

[28] Chou SH, Chang YT, Li HP, Huang MF, Lee CH, Lee KW (2010). Factors predicting the hospital mortality of patients with corrosive gastrointestinal injuries receiving esophagogastrectomy in the acute stage. World J Surg.

[29] Dapri G, Himpens J, Mouchart A, Ntounda R, Claus M, Dechamps P (2007). Laparoscopic transhiatal esophago-gastrectomy after corrosive injury. Surg Endosc.

[30] Díaz-Sánchez A, Carrión G, Barreiro A, Ortiz C, De Fuenmayor ML, Gimeno M (2001). Massive gastric necrosis from hydrochloric acid ingestion. Eur J Surg.

[31] Di Saverio S, Biscardi A, Piccinini A, Mandrioli M, Tugnoli G. Different possible surgical managements of caustic ingestion: diagnostic laparoscopy for Zargar’s grade 3a lesions and a new technique of “duodenal damage control” with “4-tube ostomy” and duodenal wash-out as an option for extensive 3b lesions in unstable patients. Updates Surg 2015: DOI 10.1007/s13304-015-0313-4 10.1007/s13304-015-0313-4 26141256

[32] Fulton JA, Hofman RS (2007). Steroids in second degree caustic burns of the esophagus: a systematic pooled analysis of fifty years of human data:1956–2006. Clin. Toxicol. (Phila.).

[33] Ganepola GA, Bhuta K (1984). A case of total esophago-gastro-duodeno-jejunectomy and partial pancreatectomy for lye burns, and reconstruction with colon interposition. J Trauma.

[34] Gupta SK, Rana AS, Gupta D, Jain G, Kalra P (2011). Unusual presentation of caustic ingestion and its surgical treatment: a case report. J Maxillofac Oral Surg.

[35] Horvath OP, Olah T, Zentai G (1991). Emergency esophagogastrectomy for treatment of hydrochloric acid injury. Ann Thorac Surg.

[36] Huscher CG, Mingoli A, Mereu A, Sgarzini G (2011). Laparoscopy can be very effective in reducing mortality rate for caustic ingestion in suicide attempt. World J Surg.

[37] Jaillard S, Nseir S, Métois D, Marquette C, Darras J, Porte H (2002). Extensive corrosive injuries of the upper airways and gastrointestinal tract. J Thorac Cardiovasc Surg.

[38] Jain RK, Gouda NB, Sharma VK, Dubey TN, Shende A, Malik R (2010). Esophageal complications following aluminium phosphide ingestion: an emerging issue among survivors of poisoning. Dysphagia.

[39] Javed A, Pal S, Krishnan E, Sanhi P, Chattopadhyay T (2012). Surgical management and outcomes of severe gastrointestinal injuries due to corrosive ingestion. World J Gastroenterol.

[40] Karagiozoglou-Lampoudi T, Agakidis CH, Chryssostomidou S, Arvanitidis K, Tsepis K (2011). Conservative management of caustic substance ingestion in a pediatric department setting, short-term and long-term outcome. Dis Esophagus.

[41] Keh SM, Onyekwelu N, McManus K, McGuigan J (2006). Corrosive injury to upper gastrointestinal tract: Still a major surgical dilemma. World J Gastroenterol.

[42] Kua K, Jonas N, O'Donnell R (2015). The larynx and caustic soda ingestion. Arch Dis Child.

[43] Lee M (2010). Caustic ingestion and upper digestive tract injury. Dig Dis Sci.

[44] Landen S, Wu MH, Jeng LB, Delugelau V, Launois B (2000). Pancreaticoduodenal necrosis due to caustic burn. Acta Chir Belg.

[45] Lefrancois M, Gaujoux S, Resche-Rigon M, Chirica M, Munoz-Bongrand SE, Cattan P (2011). Oesophagogastrectomy and pancreaticoduodenectomy for caustic injury. Br J Surg.

[46] Losanoff J, Kjossef K (1996). Multivisceral injury after liquid caustic ingestion. Surgery.

[47] Mamede RCM, De Mello Filho FV (2002). Treatment of caustic ingestion: an analysis of 239 cases. Dis Esophagus.

[48] Munoz-Bongrand N, Cattan P, de Chaisemartin C, Bothereau H, Honigman I, Sarfati E (2003). Extensive digestive caustic burns: what are the limits for resection? A series of 12 patients. Ann Chir.

[49] Ramakrishnaiah VP, Gangavatiker R, Dash NR, Sahni P (2013). Pancreas-sparing duodenectomy in a patient with acute corrosive injury. Updates Surg.

[50] Rigo GP, Camellini L, Azzolini F, Guazzetti S, Bedogni G, Merighi A (2002). What is the utility of selected clinical and endoscopic parameters in predicting the risk of death after caustic ingestion?. Endoscopy.

[51] Ryu HH, Jeung KW, Lee BK, Uhm JH, Park YH, Shin MH (2010). Caustic injury: can CT scan grading system enable prediction of esophageal stricture?. Clin Toxicol.

[52] Sarfati E, Jacob L, Servant JM, d’Acremont B, Roland E, Ghidalia T (1992). Tracheobronchial necrosis after caustic ingestion. J Thorac Cardiovasc Surg.

[53] Subasinghe D, Rathnasena BG (2011). Early laparoscopic gastrojejunostomy for corrosive injury of upper gastrointestinal tract. Trop Gastroenterol.

[54] Tohda G, Sugawa C, Gayer C, Chino A, McGuire TW, Lucas CE (2008). Clinical evaluation and management of caustic injury in the upper gastrointestinal tract in 95 adult patients in an urban medical center. Surg Endosc.

[55] Vereczkei A, Varga G, Pótó L, Horváth OP (1999). Management of corrosive injuries of the esophagus. Acta Chir Hung.

[56] Wu MH, Lai WW, Hwang TL, Lee SC, Hsu HK, Lin TS (1996). Surgical results of corrosive injuries involving esophagus to jejunum. Hepatogastroenterology.

[57] Zerbib P, Voisin B, Truant S, Saulnier F, Vinet A, Chambon JP (2011). The conservative management of severe caustic gastric injuries. Ann Surg.

[58] Baskin D, Urganci N, Abbasoğlu L, Alkim C, Yalçin M, Karadağ C (2004). A standardised protocol for the acute management of corrosive ingestion in children. Pediatr Surg Int.

[59] Berger M, Ure B, Lacher M (2012). Mitomycin C in the therapy of recurrent esophageal strictures: hype or hope?. Eur J Pediatr Surg.

[60] Betalli P, Rossi A, Bini M, Bacis G, Borrelli O, Cutrone C (2009). Update on management of caustic and foreign body ingestion in children. Diagn Ther Endosc.

[61] Bueno R, Swanson SJ, Jaklitsch MT, Lukanich JM, MentzerSJ SDJ (2001). Combined antegrade and retrogradedilation: a new endoscopic technique in the management ofcomplex esophageal obstruction. Gastrointest Endosc.

[62] Contini S, Garatti M, Swarray-Deen A, Depetris N, Cecchini S, Scarpignato C (2009). Corrosive oesophageal strictures in children: outcomes after timely or delayed dilatation. Dig Liver Dis.

[63] Contini S, Scarpignato C, Rossi A, Strada G (2011). Features and management of esophageal corrosive lesions in children in Sierra Leone: lessons learned from 175 consecutive patients. J Pediatr Surg.

[64] Contini S, Swarray-Deen A, Scarpignato C (2009). Oesophageal corrosive injuries in children: a forgotten social and health challenge in developing countries. Bull World Health Organ.

[65] Dall’Oglio L, De Angelis P (2012). Commentary on “Esophageal endoscopic dilations”. J Pediatr Gastroenterol Nutr.

[66] De Peppo F, Zaccara A, Dall’Oglio L, Federici di Abriola G, Ponticelli A, Marchetti P (1998). Stenting for caustic strictures: esophageal replacement replaced. J Pediatr Surg.

[67] Doğan Y, Erkan T, Cokuğraş FC, Kutlu T (2006). Caustic gastroesophageal lesions in childhood: an analysis of 473 cases. Clin Pediatr (Phila).

[68] Foschia F, De Angelis P, Torroni F, Romeo E, Caldaro T, diAbriola GF (2011). Custom dynamic stent for esophageal strictures in children. J Pediatr Surg.

[69] Genc A, Mutaf O (2002). Esophageal motility changes in acute and late periods of caustic esophageal burns and their relation to prognosis in children. J Pediatr Surg.

[70] Gerçek A, Ay B, Dogan V, Kiyan G, Dagli T, Gogus Y (2007). Esophageal balloon dilation in children: prospective analysis of hemodynamic changes and complications during general anesthesia. J Clin Anesth.

[71] Gumaste VV, Dave PB (1992). Ingestion of corrosive substances by adults. Am J Gastroenterol.

[72] Gün F, Abbasoğlu L, Celik A, Salman ET (2007). Early and late term management in caustic ingestion in children: a 16-year experience. Acta Chir Belg.

[73] Gundogdu HZ, Tanyel FC, Büyükpamukçu N, Hiçsönmez A (1992). Conservative treatment of caustic esophageal strictures in children. J Pediatr Surg.

[74] Ham YH, Kim GH (2014). Plastic and biodegradable stents for complex and refractory benign esophageal strictures. Clin Endosc.

[75] Karnak I, Tanyel FC, Büyükpamukçu N, Hiçsönmez A (1999). Combined use of steroid, antibiotics and early bougienage against stricture formation following caustic esophageal burns. J. Cardiovasc. Surg.

[76] Makharia GK, Kochhar R (2002). Usefulness of intralesional triamcinolone in treatment of benign esophageal strictures. Gastrointest Endosc.

[77] Kochman ML, McClave SA, Boyce HW (2005). The refractory and the recurrent esophageal stricture: a definition. Gastrointest Endosc.

[78] Kukkady A, Pease PW (2002). Long-term dilatation of caustic strictures of the oesophagus. Pediatr Surg Int.

[79] Lahoti D, Broor SL, Basu PP, Gupta A, Sharma R, Pant CS (1995). Corrosive esophageal strictures: predictors of response to endoscopic dilation. Gastrointest Endosc.

[80] Lakhdar-Idrissi M, Khabbache K, Hida M (2012). Esophageal endoscopic dilations. J Pediatr Gastroenterol Nutr.

[81] Lew RJ (2002). Kochman ML A review of endoscopic methods of esophageal dilation. J Clin Gastroenterol.

[82] Millar JW, Cox SG (2015). Caustic injuries of the esophagus. Pediatr Surg Int.

[83] Mutaf O, Genç A, Herek O, Demircan M, Ozcan C, Arikan A (1996). Gastroesophageal reflux: a determinant in the outcome of caustic esophageal burns. J Pediatr Surg.

[84] Orive-Calzada A, Bernal-Martinez A, Navajas-Laboa M, Torres-Burgos S, Aguirresarobe M, Lorenzo-Morote M (2012). Efficacy of intralesional corticosteroid injection in endoscopic treatment of esophageal strictures. Surg. Laparosc. Endosc. Percutan. Tech.

[85] Repici A, Hassan C, Sharma P, Conio M, Siersema P (2010). Systematic review: the role of self-expanding plastic stents for benign oesophageal strictures. Aliment Pharmacol Ther.

[86] Saleem MM (2009). Acquired oesophageal strictures in children: emphasis on the use of string-guided dilatations. Singapore Med J.

[87] Sandgren K, Malmfors G (1998). Balloon dilatation of oesophageal strictures in children. Eur J Pediatr Surg.

[88] Scolapio JS, Tousif M, Gostout CJ, Mahoney DW, Zinsmeister AR, Ott BJ (1999). A randomized prospective study comparing rigid to balloon dilators for benign esophageal strictures and rings. Gastroint Endosc.

[89] Shehata SM, Enaba ME (2012). Endoscopic dilatation for benign oesophageal strictures in infants and toddlers: experience of an expectant protocol from North African tertiary centre. Afr J Paediatr Surg.

[90] Spechler SJ (1999). American gastroenterological association medical position statement on treatment of patients with dysphagia caused by benign disorders of the distal esophagus. Gastroenterology.

[91] Temiz A, Oguzkurt P, Ezer SS, Ince E, Hicsonmez A (2010). Long-term management of corrosive esophageal stricture with balloon dilation in children. Surg Endosc.

[92] Tiryaki T, Livanelioğlu Z, Atayurt H (2005). Early bougienage for relief of stricture formation following caustic esophageal burns. Pediatr Surg Int.

[93] van Halsema EE, van Hooft JE (2015). Clinical outcomes of self-expandable stent placement for benign esophageal diseases: A pooled analysis of the literature. World J Gastrointest Endosc.

[94] Vimalraj V, Rajendran S, Jyotibasu D, Balachandar TG, Kannan D, Jeswanth S (2007). Role of retrograde dilatation in the management of pharyngo-esophageal corrosive strictures. Dis Esophagus.

[95] Wang RW, Zhou JH, Jiang YG, Fan SZ, Gong TQ, Zhao YPTan QY (2006). Prevention of stricture with intraluminal stenting through laparotomy after corrosive esophageal burns. Eur J Cardiothorac Surg.

[96] Wijburg FA, Heymans HS, Urbanus NA (1989). Caustic esophageal lesions in childhood: prevention of stricture formation. J Pediatr Surg.

[97] Lerut T, Mbamendame S, Touré BM, Coulibaly Z, Kané B, Diani N (2014). Les sténoses caustiques de l’oesophage à l’hôpital du Mali. Ampleur, gravité et place de la dilatation en chirurgie. Chir Thorac Cardio-Vascul.

[98] Zargar SA, Kochhar R, Mehta S, Mehta SK (1991). The role of fiberoptic endoscopy in the management of corrosive ingestion and modified endoscopic classification of burns. Gastrointest Endosc.

[99] Abaskharoun RD, Depew WT, Hookey LC (2007). Nonsurgical management of severe esophageal and gastric injury following alkali ingestion. Can J Gastroenterol.

[100] Agarwal S, Sikora SS, Kumar A, Saxena R, Kapoor VK (2004). Surgical management of corrosive strictures of stomach. Indian J Gastroenterol.

[101] Ananthakrishnan N, Parthasarathy G, Kate V (2010). Chronic corrosive injuries of the stomach-a single unit experience of 109 patients over thirty years. World J Surg.

[102] Ansari MM, Haleem S, Harris SH, Khan R, Zia I, Beg MH (2011). Isolated corrosive pyloric stenosis without oesophageal involvement: an experience of 21 years. Arab J Gastroenterol.

[103] Chaudhary A, Puri AS, Dhar P, Reddy P, Sachdev A, Lahoti D (1996). Elective surgery for corrosive-induced gastric injury. World J Surg.

[104] Chibishev A, Pereska Z, Simonovska N, Chibisheva V, Glasnovic M, Chitkushev LT (2013). Conservative therapeutic approach to corrosive poisonings in adults. J Gastrointest Surg.

[105] Tai WC, Chiu KW, Chuah SK, Chiu YC, Liang CM, Tam W (2013). The effects of endoscopic-guided balloon dilations in esophageal and gastric strictures caused by corrosive injuries. BMC Gastroenterol.

[106] Gupta V, Wig JD, Kochhar R, Sinha SK, Nagi B, Doley RP (2009). Surgical management of gastric cicatrisation resulting from corrosive ingestion. Int J Surg.

[107] Hogan RB, Polter DE (1986). Non surgical management of lye-induced antral strictures with hydrostatic balloon dilation. Gastrointest Endosc.

[108] Hwang TL, Chen MF (1996). Surgical treatment of gastric outlet obstruction after corrosive injury—can early definitive operation be used instead of staged operation?. Int Surg.

[109] Kaushik R, Singh R, Sharma R, Attri AK, Bawa AS (2003). Corrosive-induced gastric outlet obstruction. Yonsei Med J.

[110] Kochhar R, Dutta U, Sethy PK, Singh G, Sinha SK, Nagi B (2009). Endoscopic balloon dilation in caustic-induced chronic gastric outlet obstruction. Gastrointest Endosc.

[111] Kochhar R, Poornachandra KS, Dutta U, Agrawal A, Singh K (2010). Early endoscopic balloon dilation in caustic-induced gastric injury. Gastrointest Endosc.

[112] Kochhar R, Sethy PK, Nagi B, Wig JD (2004). Endoscopic balloon dilatation of benign gastric outlet obstruction. J Gastroenterol Hepatol.

[113] Lebeau R, Coulibaly A, Kountélé Gona S, Koffi Gnangoran M, Kouakou B, Yapo P (2011). Isolated gastric outlet obstruction due to corrosive ingestion. J Visc Surg.

[114] Lu LS, Tai WC, Hu ML, Wu KL, Chiu YC. Predicting the progress of caustic injury to complicated gastric outlet obstruction and esophageal stricture, using modified endoscopic mucosal injury grading scale. Biomed Research International 2014:919870. doi:10.1155/2014/919870. Epub 2014 Aug 4. 10.1155/2014/919870 PMC4137736 25162035

[115] Manta R, Conigliaro R, Bertani H, Manno M, Soliman A, Fedeli P, Bassotti G. Self-Expandable Metal Stenting of Refractory Upper Gut Corrosive Strictures: A New Role for Endoscopy? Case Reports in Gastrointestinal Medicine 2011, doi:10.1155/2011/346413 10.1155/2011/346413 PMC3350195 22606415

[116] Rana SS, Bhasin DK, Chandail VS, Gupta R, Nada R, Kang M (2011). Endoscopic balloon dilatation without fluoroscopy for treating gastric outlet obstruction because of benign etiologies. Surg Endosc.

[117] Solt J, Bajor J, Szabó M, Horváth OP (2003). Long-term results of balloon catheter dilation for benign gastric outlet stenosis. Endoscopy.

[118] Tseng YL, Wu MH, Lin MY, Lai WW (2002). Early surgical correction for isolated gastric stricture following acid corrosion injury. Dig Surg.

[119] Bonavina L, Chella B, Segalin A, Luzzani S (1998). Surgical treatment of the redundant interposed colon after retrosternal esophagoplasty. Ann Thorac Surg.

[120] Bothereau H, Munoz-Bongrand N, Lambert B, Montemagno S, Cattan P, Sarfati E (2007). Esophageal reconstruction after caustic injury: is there still a place for right coloplasty?. Am J Surg.

[121] Boukerrouche A (2013). Left colonic graft in esophageal reconstruction for caustic stricture: mortality and morbidity. Dis Esoph.

[122] Chana JS, Chen HC, Sharma R, Gedebou TM, Feng GM (2002). Microsurgical reconstruction of the esophagus using supercharged pedicled jejunum flaps: special indications and pitfalls. Plast Reconstr Surg.

[123] Cheng YJ, Wang KU, Chen HC, Hsieh KC, Chang PC (2010). Esophageal mucocele with compression of the right recurrent laryngeal nerve 20 years after surgical intervention for caustic esophagitis. Ann Thorac Surg.

[124] Chirica M, Veyrie N, Munoz-Bongrand N, Zohar S, Halimi B, Celerier M (2010). Late morbidity after colon interposition for corrosive esophageal injury. Risk factors, management and outcome. A 20-years experience. Ann Surg.

[125] Deng B, Wang RW, Jiang YG, Gong TQ, Zhou JH, Lin YD (2008). Prevention and management of complications after colon interposition for corrosive esophageal burns. Dis Esophagus.

[126] Evans KFK, Mardini S, Salgado CJ, Chen HC (2010). Esophagus and hypopharyngeal reconstruction. Semin Plast Surg.

[127] Ezemba N, Eze JC, Nwafor IA, Etukokwu KC, Orakwe OI (2014). Colon interposition graft for corrosive esophageal stricture: midterm functional outcome. World J Surg.

[128] Gerzic ZB, Knezevic JB, Milicevic MN, Jovanovic BK (1990). Esophagocoloplasty in the management of postcorrosive strictures of the esophagus. Ann Surg.

[129] Guha G, Gupta S, Chakraborty S (2005). Pharyngo-oesophageal strictures and its reconstruction by delto-pectoral flaps. Indian J Otolaryngol Head Neck Surg.

[130] Gupta S (1996). Surgical management of corrosive strictures following acid burns of upper gastrointestinal tract. Eur J Cardiothorac Surg.

[131] Gupta NM, Gupta R (2004). Transhiatal esophageal resection for corrosive injury. Ann Surg.

[132] Han Y, Cheng QS, Li XF, Wang XP (2004). Surgical management of esophageal strictures after caustic burns: A 30 years of experience. World J Gastroenterol.

[133] Harlak A, Yigit T, Coskun K, Ozer T, Mentes O, Gulec B (2013). Surgical treatment of caustic esophageal strictures in adults. Int J Surg.

[134] Ho AC, Yeo MS, Ciudad P, Chen HC (2014). 2-Stage free and pedicle jejunum for esophageal replacement after failed colon interposition for caustic injury in a 5 year-old child. J Plast Reconstr Aesthet Surg.

[135] Javed A, Pal S, Dash NR, Sahni P, Chattopadhyay TK (2011). Outcome following surgical management of corrosive strictures of the esophagus. Ann Surg.

[136] Jiang YG, Lin YD, Wang RW, Zhou JH, Gong TQ, Ma Z (2005). Pharyngocolonic anastomosis for esophageal reconstruction in corrosive esophageal stricture. Ann Thorac Surg.

[137] Kane TD, Nwomeh BC, Nadler EP (2007). Thoracoscopic-assisted esophagectomy and laparoscopic gastric pull-up for lye injury. JSLS.

[138] Khwa-Otsyula BO, Matthews HR, Shenoi PM (1993). Laryngectomy and coloplasty for major oesophagogastric corrosive stricture: case report and literature review. East Afr Med J.

[139] Kim YT, Sung SW, Kim JH (2001). Is it necessary to resect the diseased esophagus in performing reconstruction for corrosive esophageal stricture?. Eur J Cardiothorac Surg.

[140] Liu HP, Chang CH, Lin PJ, Chang JP (1995). Video-assisted endoscopic esophagectomy with stapled intrathoracic esophagogastric anastomosis. World J Surg.

[141] Nwomeh BC, Luketich JP, Kane TD (2004). Minimally invasive esophagectomy for caustic esophageal stricture in children. J Pediatr Surg.

[142] Park JK, Sim SB, Lee SH, Jeon HM, Kwack MS (2001). Pharyngo-enteral anastomosis for esophageal reconstruction in diffuse corrosive esophageal stricture. Ann Thorac Surg.

[143] Popovici ZA (2003). new philosophy in esophageal reconstruction with colon. Thirty-years experience. Dis Esophagus.

[144] Radovanović N, Simić A, Kotarac M, Stojakov D, Sabljak P, Skrobić O (2009). Colon interposition for pharyngoesophageal postcorrosive strictures. Hepatogastroenterology.

[145] Ribet M, Chambon JP, Pruvot FR (1990). Oesophagectomy for severe corrosive injuries: is it always legitimate?. Eur J Cardiothorac Surg.

[146] Sa YJ, Kim YD, Kim CK, Park JK, Moon SW (2013). Recurrent cervical esophageal stenosis after colon conduit failure: use of myocutaneous flap. World J Gastroenterol.

[147] Saetti R, Silvestrini M, Cutrone C, Barion U, Mirri L, Narne S (2003). Endoscopic treatment of upper airway and digestive tract lesions caused by caustic agents. Ann Otol Rhinol Laryngol.

[148] Spitz L, Kiely E, Pierro A (2004). Gastric transposition in children - A 21-year experience. J Pediatr Surg.

[149] Tettey M, Edwin F, Aniteye E, Tamatey M, Entsua-Mensah K, Ofosu-Appiah E (2011). Colopharyngoplasty for intractable caustic pharyngoesophageal strictures in an indigenous African community – adverse impact of concomitant tracheostomy on outcome. Interact Cardiovasc Thorac Surg.

[150] Pungpapong SA, Udomsawaengsup S, Navicharern P (2010). Thoracoscopic approach for esophageal resection in chronic severe corrosive esophageal stricture: report of 2 cases. J Med Assoc Thai.

[151] Yannopoulos P, Lytras D, Paraskevas KI (2013). Esophageal reconstruction with intraoperative dilatation of the hypopharynx for the management of chronic corrosive esophageal strictures. A technical tip. Eur J Cardiothorac Surg. 2006;30(6):940–2.Javed A, Agarwal AK. Total laparoscopic esophageal bypass using a colonic conduit for corrosive-induced esophageal stricture. Surg Endosc.

[152] Yasuda T, Shiozaki H (2011). Esophageal reconstruction using a pedicled jejunum with microvascular augmentation. Ann Thorac Cardiovasc Surg.

[153] Yildirim S, Koksal H, Celayir T, Erdem L, Oner M, Baykan A (2004). Colonic interposition vs. gastric pull-up after total esophagectomy. J Gastrointest Surg.

[154] Wu MH, Lai WW (1992). Esophageal reconstruction for esophageal strictures or resection after corrosive injury. Ann Thorac Surg.

[155] Wu MH, Tseng YL, Lin MY, Lai WW (2001). Esophageal reconstruction for hypopharyngoesophageal strictures after corrosive injury. Eur J Cardio-Thorac Surg.

[156] Zhou JH, Jiang YG, Wang RW, Lin YD, Gong TQ, Zhao YP (2005). Management of corrosive esophageal burns in 149 cases. J Thorac Cardiovasc Surg.

[157] Anderson KD, Rouse TM, Randolph JG (1990). A controlled trial of corticosteroids in children with corrosive injury of the esophagus. N Engl J Med.

[158] Kluger Y et al. Caustic ingestion management: World Society of Emergency Surgery preliminary survey of expert opinion. World J Emerg Surg 2015 (in press). 10.1186/s13017-015-0043-4 PMC4609064 26478740

